# *In Vitro* Control of Post-Harvest Fruit Rot Fungi by Some Plant Essential Oil Components

**DOI:** 10.3390/ijms13022290

**Published:** 2012-02-21

**Authors:** Ippolito Camele, Luciana Altieri, Laura De Martino, Vincenzo De Feo, Emilia Mancini, Gian Luigi Rana

**Affiliations:** 1 Department of Biology, Plant Protection and Agro-Forestry Biotechnology, Basilicata University, Viale dell’Ateneo Lucano, 10-85100, Potenza, Italy; E-Mails: ippolito.camele@unibas.it (I.C.); Luciana.altieri@unibas.it (L.A.); gianluigi.rana@unibas.it (G.L.R.);; 2 Department of Pharmaceutical and Biomedical Sciences, Salerno University, Fisciano, Salerno, Italy; E-Mails: ldemartino@unisa.it (L.D.M.); emancini@unisa.it (E.M.)

**Keywords:** fungitoxic activity, monoterpenes, plant essential oils, post-harvest diseases

## Abstract

Eight substances that are main components of the essential oils from three Mediterranean aromatic plants (*Verbena officinalis*, *Thymus vulgaris* and *Origanum vulgare*), previously found active against some phytopathogenic Fungi and Stramenopila, have been tested *in vitro* against five etiological agents of post-harvest fruit decay, *Botrytis cinerea*, *Penicillium italicum*, *P. expansum*, *Phytophthora citrophthora* and *Rhizopus stolonifer*. The tested compounds were β-fellandrene, β-pinene, camphene, carvacrol, citral, *o*-cymene, γ-terpinene and thymol. Citral exhibited a fungicidal action against *P. citrophthora*; carvacrol and thymol showed a fungistatic activity against *P. citrophthora* and *R. stolonifer*. Citral and carvacrol at 250 ppm, and thymol at 150 and 250 ppm stopped the growth of *B. cinerea*. Moreover, thymol showed fungistatic and fungicidal action against *P. italicum*. Finally, the mycelium growth of *P. expansum* was inhibited in the presence of 250 ppm of thymol and carvacrol. These results represent an important step toward the goal to use some essential oils or their components as natural preservatives for fruits and foodstuffs, due to their safety for consumer healthy and positive effect on shelf life extension of agricultural fresh products.

## 1. Introduction

The most important plant pathogens, *i.e.*, fungi (*s.l*.), bacteria, phytoplasmas and viruses, can cause considerable economic damages to plant products. Among them, fungi (*s.l*.) constitute the more numerous group of plant pathogens and can often cause severe diseases in vegetable and fruit species [1]. Over several decades, various attempts have been accomplished to prevent, control, or eradicate plant diseases, and development of synthetic fungicides was particularly investigated [2]. These pesticides are known to be highly effective in controlling various postharvest diseases of vegetables and fruits. Although effective, their continued or repeated applications may disrupt equilibrium of ecosystems, leading to dramatic disease outbreaks, widespread development of pathogens resistant to one or more chemicals, toxicity to non-target organisms and environmental problems [2]. Sometimes, they accumulate in the food chain as residues above safe limits [3]. Furthermore, pesticide residues in food possess more carcinogenic risks than insecticides and herbicides [2]. A noticed decrease in pesticide efficacy, along with increased concern about the environmental effects of currently used fungicides as well as dramatic reduction of the marketable ones due to recent European laws, have highlighted the need to develop alternative control strategies or innovative crop protection and postharvest methods of fruit rot control with reduced use of conventional fungicides or without synthetic chemicals at all [4].

Research on plant-derived fungicides is now being intensified, as it became evident that these substances have enormous potential to improve the future agrochemical technology. In fact, there are good reasons to suppose that secondary plant metabolism has naturally evolved to actively protect vegetable and fruit species from microbial pathogen attacks [4]. Since secondary plant metabolites are often active against a small number of specific target microorganism species and are biodegradable to nontoxic products, they are potentially useful in integrated pest management programs: moreover, they could allow to develop a new class of possibly safer disease control substances. Therefore, efforts have been focused on secondary plant metabolites for their potential use as commercial fungicides or as lead compounds [5].

Among secondary plant metabolites, plant essential oils (PEOs) may provide potential alternatives to active substances currently used to control phytopathogenic fungi (*s.l*.), since they are not only a fragrance and flavour source for food and beverages but are also being discovered as bioactive substances tanks [6].

Several investigations have been carried out abroad in this field toward antimicrobial and fungicidal PEOs properties [3,7–11]. In particular, PEOs use to control postharvest fruit and vegetable diseases have been studied and are nowadays well documented [12–15]. In Italy, in addition to research carried out by Conte *et al*. [16] and Zambonelli *et al*. [17], more recently studies on the potential fungicidal activity of 12 PEOs from Mediterranean aromatic plants against five postharvest fungal (*s.l.*) pathogens of orange fruits, were accomplished by our research group [18]. Results of this research showed that PEOs from *Verbena officinalis* L., *Thymus vulgaris* L. and *Origanum vulgare* L. could be used as a possible source of ecofriendly botanical fungicides for controlling important postharvest fungal pathogens without phytotoxicity risk. Furthermore, it seemed believable that PEO volatile components could be employed to uniformly permeate storeroom air and protect, maybe better than fungicides commonly used for this goal, fruits that are in storage. Therefore, it seemed opportune to assay the *in vitro* fungicidal activity of the above mentioned PEO main components against five fungi (*s.l*.) well known as postharvest fruit decaying agents.

## 2. Results and Discussion

**Figure 1:**
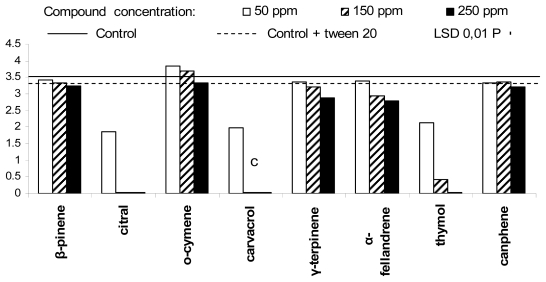
Antifungal activity of the eight plant essential oil components against *Phytophthora citrophthora.*

**Figure 2:**
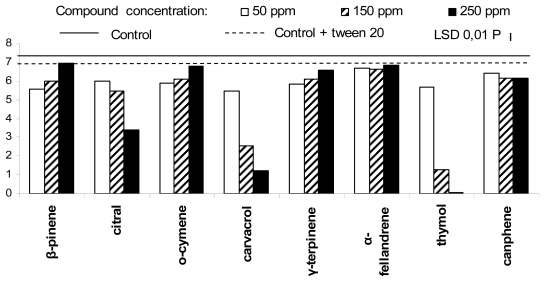
Antifungal activity of the eight plant essential oil components against *Rhizopus stolonifer*.

**Figure 3:**
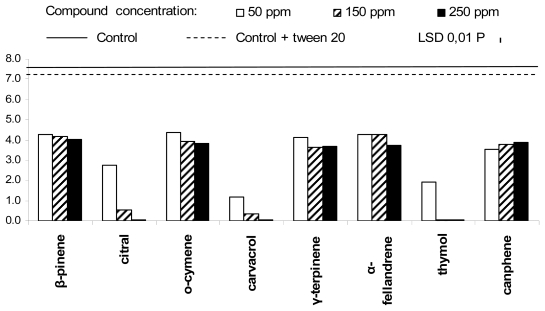
Antifungal activity of the eight plant essential oil components against *Botrytis cinerea.*

**Figure 4:**
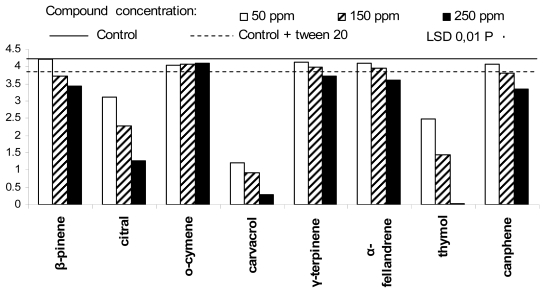
Antifungal activity of the eight plant essential oil components against *Penicillium italicum*.

**Figure 5:**
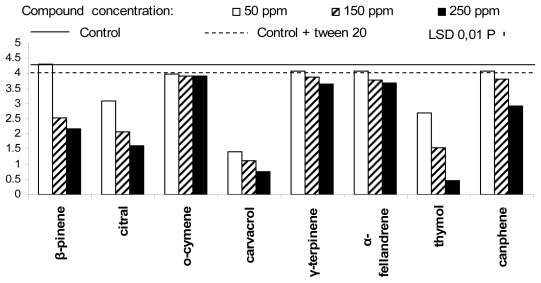
Antifungal activity of the eight plant essential oil components against *Penicillium expansum*.

The *in vitro* activity of the tested compounds against the five post-harvest fruit decaying agents, registered ten days after experiment beginning, is summarized in Figures 1–5. In particular, *Phytophthora citrophthora* did not show any mycelium growth in presence of citral and carvacrol, at concentration of 150 and 250 ppm, and in presence of thymol, at 250 ppm (Figure 1). The same organism showed a reduced growth in presence of thymol, at 150 ppm, and citral, carvacrol and thymol, at 50 ppm, and grew more or less weakly in presence of β-fellandrene and γ-terpinene at 150 and 250 ppm. Surprisingly, *o*-cymene, at 50 and 150 ppm, promoted a more rapid growth of the above Oomycete, in comparison to controls.

The growth of *Rhizopus stolonifer* appeared completely inhibited by thymol, at 250 ppm (Figure 2), and reduced in presence of 150 ppm thymol, 150 and 250 ppm carvacrol and 250 ppm citral. The same microorganism seemed less affected by the other PEO components. The mycelium growth of *Botrytis cinerea* (Figure 3) was totally inhibited by citral and carvacrol, at concentration of 250 ppm, and by thymol, at 150 and 250 ppm. Carvacrol dramatically inhibited the mycelium growth of the same fungus even at 50 ppm, whereas thymol and citral, at the lower used concentration, were significantly less effective. In general, *B. cinerea* was well controlled by all the other PEO components independently of the concentration used.

The growth of *Penicillium italicum* (Figure 4) was completely blocked by thymol at 250 ppm concentration, more or less satisfactorily controlled by the three carvacrol concentrations and appeared reduced in presence of 250 ppm citral. *P. italicum* was not affected in its growth by β-pinene at 50 ppm; at the opposite, it showed only a weak colony extension (0.8 cm in average) in Petri dishes containing 150 ppm thymol. Its mycelium appeared white in colour and completely lacking of conidiophore and conidia, when observed at light microscope. The same fungus showed scanty conidiophores production and almost none conidium differentiation in Petri dishes containing citral, at 150 ppm concentration. The growth of *Penicillium expansum* (Figure 5), appeared greatly reduced by thymol at 250 ppm, significantly loosened by all three carvacrol concentrations and by citral and thymol, at 250 and 150 ppm, respectively. The same fungus did not produce conidiophores and conidia in presence of thymol (150 and 250 ppm), or carvacrol (250 ppm); like the other *Penicillium* species, *P. expansum* appeared not to be affected by 50 ppm β-pinene.

Tested phytopathogenic microorganisms grew with the expected rapidity in control plates and significantly less rapidly, except for *R. stolonifer*, in those containing Tween-20. Colony diameter in control plates reached its maximum length in 4–10 days.

Among microorganisms which did not show any mycelium growth in presence of some PEO components, only *P. citrophthora* (exposed to 150 and 250 ppm carvacrol), *R. stolonifer* (which seemed killed by 250 ppm thymol) and *B. cinerea*, (which seemed killed by citral and carvacrol, at 250 ppm, and thymol at 150 and 250 ppm) showed a weak colony growth four days after the respective corn meal agar (CMA) and potato dextrose agar (PDA) plugs were transferred from Petri dishes on CMA or PDA. This demonstrated that they were still alive although subjected for some days to exposure to the above PEO components.

Conversely, *P. citrophthora*, exposed to 150 and 250 ppm of citral, and to 250 ppm of thymol, and *P. italicum*, in presence of thymol at 250 ppm, did not show any micelium growth after being transferred on CMA or PDA, respectively. This demonstrated that these two microorganisms have been effectively killed by the higher concentrations of tested compounds. Colony growth of the last two microorganisms from PDA plugs and CMA plugs exposed to the lower PEO component concentrations was rather slow and then did not differ from that observed in control colonies. All tested microorganisms from basic colonies grew normally in control plates containing only PDA or CMA.

PEO efficacy against postharvest fruit decaying fungi (*s.l*.) is thought to depend on specific toxicity of their single main active constituents or by their synergic effect [15]. Our results show that some of the PEO constituents, even if used at very low concentrations, exhibited *in vitro* a fungistatic and/or, in some instances, a fungicidal action against the tested organisms and, therefore, could be employed to control food-stuff decaying agents. In fact, among the eight compounds tested, only β-pinene is reported as toxic for humans [19,20]. Particularly. carvacrol, citral and thymol appear as a promising candidates for future *in vivo* tests. The last two compounds resulted fungicides against *P. citrophthora*, whereas thymol at 250 ppm killed *P. italicum* and markedly inhibited mycelium growth of *P. expansum*, the well-known and dangerous patulin producer.

The available literature reported PEOs as active drugs against phytopathogenic fungi, but very few researches demonstrated the possible fungitoxicity of their constituents. In particular, γ-terpinene, assayed *in vitro* in gaseous state, showed a strong inhibitory activity against *B. cinerea* [21]. Camphene was reported for its antifungal activity against five phytopathogenic fungi [22]. Carvacrol and thymol at 100 ppm, completely inhibited mycelial growth of several phytopathogenic fungi [23]. The strong antifungal activity of carvacrol and thymol (100% of inhibition against *B. cinerea*) was also reported [24,25]. Mueller-Riebau and coworkers [26] showed that fungitoxicity against the soil-borne plant disease-causing fungi *Fusarium moniliforme* J. Sheld., *Rhizoctonia solani* J.G. Kühn, *Sclerotinia sclerotiorum* (Lib.) de Bary and *Phytophthora capsici* Leon. was due to different concentrations of the phenolic fraction (thymol and/or carvacrol) present in the PEOs different aromatic plants. These two compounds were also reported for their *in vitro* antifungal activity against *Ophiostoma novo-ulmi* Brasier, causal agent of Dutch elm disease [27].

Moreover, recently, Soković and coworkers [28] reported a relationship between the high activity of some *Thymus* oils and the presence of phenol components, such as thymol and carvacrol. The high antifungal activity of these essential oils could be explained by the high percentage of phenol components. It seems possible that phenol components may interfere with cell wall enzymes like chitin synthase/chitinase as well as with the α- and β-glucanases of the fungus.

Carvacrol, well known for its important fungitoxic activity against *B. cinerea* [29,30] showed non selective activity against three *Colletotrichum* strains [31] and against the major fungal pathogens of the button mushroom *Agaricus bisporus* (J.E. Lange) Imbach var. *bisporus* [32]. *Mycogone perniciosa* (Magnus) Delacr., another pathogen of this mushroom, was affected by thymol [33]. The same compound inhibited the mycelial growth of *Colletotrichum acutatum* J. H. Simmonds, which is considered as responsible for the major postharvest tomato tree disease in Colombia [34]. Thymol was reported active also against *P. expansum* and *B. cinerea* [9,35].

In our assays, citral resulted as one of more active compounds. In literature, it has been reported for its good antimicrobial activity against *P. italicum* [36], *Aspergillus niger* Tiegh. and *R. stolonifer* [37], *B. cinerea* [1,25] and *Colletotrichum acutatum* [34]. Inhibitory activity of citral or of its vapour against several phytopathogenic fungi has been repeatedly reported [38–40]. Luo and co-workers [41] suggested a possible mechanism of citral action against *Aspergillus flavus* Link: after it penetrates cell wall, irreversibly damages plasma membrane and DNA with consequent spore loss germination. Also in *A. niger*, the primary citral site action seems to be cell membrane [37]. Moreover, citral was shown able of forming charge transfer complexes with tryptophan, a good electron donor. Apparently, the antifungal actions of the aldehydes, as citral, are due to their abilities to form charge transfer complexes with electron donors in addition to their reactivity with SH groups [42].

*o*-Cymene was reported to inhibit growth of the root pathogenic fungi *Phytophthora cinnamomi* Rands and *Fomes annosus* (Fr.) Cooke [43].

## 3. Experimental Section

### 3.1. Chemicals

β-Fellandrene, β-pinene, camphene, carvacrol, citral, *o*-cymene, γ-terpinene and thymol were purchased from Sigma Aldrich, Co. (Milan, Italy). All the above compounds were kept at −20 °C.

### 3.2. Fungal and Stramenopilus Isolates

The species of plant pathogenic Fungi and Stramenopila and relative isolates used in this study and hereafter listed, were derived from a pure culture-maintained collection of the mycotheca of Department of Biology, Plant Protection and Agro-Forestry Biotechnology, Basilicata University (Potenza, Italy), kept on potato destrose agar (PDA) or corn meal agar (CMA) at 4 °C: *Phytophthora citrophthora* (R.E. & E.H. Smith) Leonian, isolate number 22 from lemon, *Botryotinia fuckeliana* (de Bary) Whetzel, used as its anamorph *Botrytis cinerea* Pers., isolate number 234 from grapevine; *Penicillium italicum* Wehrner, isolate number 333 from orange, *Penicillium expansum* Link, isolate number 335 from apple and *Rhizopus stolonifer* (Ehrenb.) Vuill., isolate number 238 from orange, were employed as basic 7–10-day-old colonies which were grown on PDA for *P. italicum*, *P. expansum*, *B. fuckeliana*, and *R. stolonifer* and on CMA, in the case of *P. citrophthora*.

### 3.3. *In Vitro* Tests

The possible fungistatic or fungicidal activity of the single PEO components against (a) *P. citrophthora*, *B. cinerea* and *R. stolonifer* and (b) *P. italicum* and *P. expansum* was determined in two ways. (a) By putting single 3-mm-thick and 0.5-cm-diameter CMA or PDA plugs, axenically taken away from peripheral portion of basic colonies onto central part surface of 9 cm diameter Petri dishes containing PDA or CMA previously added, at 40 °C, of 0.2% Tween-20 and 50, 150 or 250 ppm of the single PEO components; (b) By dropping, under axenic conditions, 10 μL aliquots of single suspensions containing 1 × 10^4^ conidia/mL of the single *Penicillium* species onto surface centre of Petri dishes containing PDA and prepared with the same percentage of Tween-20 and the single three PEO component concentration. Three replicates for each tested PEO active compound dose were provided in presence of opportune controls, *i.e.*, PDA and CMA plugs from basic colonies of *P. citrophthora*, *B. cinerea* and *R. stolonifer* and of the two *Penicillium* species transferred or dropped, respectively, as above into Petri dishes containing either sole PDA or CMA or PDA or CMA added of 0.2% tween 20.

The inhibitory effects of PEO components against each tested microorganism belonging to Stramenopila or Fungi kingdoms was determined after a 3–10 days incubation period at room temperature, e.g., when control colonies margins reached plate edges. The shortest and longest colony diameters were measured for each fungal or stramenopilus culture and the average value obtained, considered as a growth index, was subjected to statistical analysis.

### 3.4. Evaluation of Fungicidal Effect of Some PEO Components

PDA plugs of *B. cinerea* and *R. stolonifer* and CMA plugs of *P. citrophthora* from Petri dishes, prepared with PEO compound concentrations which seemed to have completely inhibited growth of tested microorganism isolates, were axenically transferred into Petri dishes containing only PDA, in the case of the first two fungi or CMA for *P. citrophthora*. Similarly, plugs axenically excised from central portion of dishes in which growth of *P. expansum* and *P. italicum* seemed to have been stopped by some PEO component concentrations, were in the same way put on PDA. Inoculated plates were then transferred in a climatic chamber at 20 ± 1 °C, along with the opportune controls, *i.e.*, Petri dishes containing PDA or CMA inoculated with agar plugs taken from basic colonies of the above phytopathogenic microorganisms. All plates were daily observed for 30 days to ascertain eventual mycelium growth.

### 3.5. Statistical Analysis

Data were statistically processed and subjected to analysis of variance (ANOVA). Means significantly different were separated by the least significant difference (LDS) with *p* < 0.01.

## 4. Conclusions

The achieved results, along with fact that essential oil poisoning is documented for a few of these volatile compounds often inappropriately used [19], constitute an interesting knowledge acquirement on the perspective of using PEOs as natural preservatives for food-stuff and stimulate to undertake *in vivo* studies to verify the possible phytotoxic effects of the above PEO active compounds and to set up an application method able to maximize their effect against target post-harvest rot causal agents [15]. On the other hand, it is important to consider that the intense smell of the PEOs’ components can limit their use in foods; in fact their perception by the consumer will stimulate technological studies in order to solve this problem.

## Abbreviations

PEOs: plant essential oils
PDA: potato dextrose agar
CMA: corn meal agar

## References

[1] Chang H.-T., Cheng Y.-H., Wua C.-L., Chang S.-T., Chang T.-T., Su Y.-C. (2008). Antifungal activity of essential oil and its constituents from *Calocedrus macrolepis* var. *formosana* Florin leaf against plant pathogenic fungi. Bioresource Technol.

[2] Lee Y.-S., Kim J., Lee S.-G., Oh E., Shin S.-C., Park I.-K. (2009). Effects of plant essential oils and components from Oriental sweetgum (*Liquidambar orientalis*) on growth and morphogenesis of three phytopathogenic fungi. Pestic. Biochem. Phys.

[3] Lee Y.-S., Kim J., Shin S.-C., Lee S.-G., Park I.-K. (2008). Antifungal activity of Myrtaceae essential oils and their components against three phytopathogenic fungi. Flavour Fragr. J.

[4] Kim M.-K., Choi G.-J., Lee H.-S. (2003). Fungicidal Property of *Curcuma longa* L. Rhizome-Derived Curcumin against Phytopathogenic Fungi in a Greenhouse. J. Agric. Food Chem.

[5] Lee H.-S., Lee S.-W., Cho K.-Y., Kim M.-K., Ahn Y.-J. (2001). Fungicidal activities of 51 fruit extracts against six phytopathogenic fungi. Agric. Chem. Biotechnol.

[6] Isman M.B. (2000). Plant essential oils for pest and disease management. Crop Prot.

[7] Plotto S., Roberts D.D., Roberts R.G. (2003). Evolution of plant essential oil as natural postharvest disease control of tomato (*Lycopersicon esculentum*). Acta Hortic.

[8] Lee S.-O., Park I.-K., Choi G.-J., Lim H.-K., Jang K.-S., Cho K.-Y., Shin S.-C., Kim J.-C. (2007). Fumigant activity of essential oils and components of *Illicium verum* and *Schizonepeta tenuifolia* against *Botrytis cinerea* and *Colletotrichum gloeosporioides*. J. Microbiol. Biotechnol.

[9] Alilou H., Akssira M., Hassani L.I., Chebli B., El Hakmoui A., Mellouki F., Rouhi R., Boira H., Blázquez M.A. (2008). Chemical composition and antifungal activity of *Bubonium imbricatum* volatile oil. Phytopathol. Med.

[10] Kim J., Lee Y.-S., Lee S.-G., Shin S.-C., Park I.-K. (2008). Fumigant antifungal activity of plant essential oils and components from West Indian bay (*Pimenta racemosa*) and thyme (*Thymus vulgaris*) oils against two phytopathogenic fungi. Flavour Fragr. J.

[11] Bajpai V.K., Lee T.J., Kang S.C. (2009). Chemical composition and *in vitro* control of agricultural plant pathogens by the essential oil and various extracts of *Nandina domestica* Thunb. J. Sci. Food Agric.

[12] Bhaskara-Reddy M.V., Angers P., Gosselin A., Arul J. (1998). Characterization and use of essential oil from *Thymus vulgaris* against *Botrytis cinerea* and *Rhyzopus stolonifer* in strawberry fruits. Phytochemistry.

[13] Chebli B., Hmamouchi M., Achouri M., Hassani L.M.I. (2004). Composition and *in vitro* fungitoxic activity of 19 essential oils against two postharvest pathogens. J. Essent. Oil Res.

[14] Tzortzakis N.G. (2007). Maintaining postharvest quality of fresh produce with volatile compounds. Innovat. Food Sci. Emerg. Technol.

[15] Lopez-Reyes J.G., Spadaro D., Gullino M.L., Garibaldi A. (2010). Efficacy of plant essential oils on postharvest control of rot caused by fungi on four cultivars of apples *in vivo*. Flavour Fragr. J.

[16] Conte A., Speranza B.S., Sinigaglia M., Deinobile M.A. (2007). Effect of lemon extract on food borne microorganisms. J. Food Protect.

[17] Zambonelli A.D., Aulerio A.Z., Bianchi A., Albasini A. (2008). Effect of essential oils on phytopathogenic fungi *in vitro*. J. Phytopathol.

[18] Camele I., De Feo V., Altieri L., Mancini E., De Martino L., Rana G.L. (2010). An Attempt of Postharvest Orange Fruit Rot Control Using Essential Oils from Mediterranean Plants. J. Med. Food.

[19] Woolf A. (1999). Essential oils poisoning. Clin. Toxicol.

[20] Landelle C., Francony G., Sam-Lai N.F., Gaillard Y., Vincent F., Wrobeleski I., Danel V. (2008). Poisoning by lavandin extract in a 18-month-old boy. Clin. Toxicol.

[21] Espinosa-Garcia F.J., Langenheim J.H. (1991). Effects of sabinene and γ-terpinene from coastal redwood leaves acting singly or in mixtures on the growth of some of their fungus endophytes. Biochem. Syst. Ecol.

[22] Pitarokili D., Tzakou O., Loukis A. (2008). Composition of the essential oil of spontaneous *Rosmarinus officinalis* from Greece and antifungal activity against phytopathogenic fungi. J. Essent. Oil Res.

[23] Kordali S., Cakir A., Ozer H., Cakmakci R., Kesdek M., Mete E. (2008). Antifungal, phytotoxic and insecticidal properties of essential oil isolated from Turkish *Origanum acutidens* and its three components, carvacrol, thymol and *p*-cymene. Bioresource Technol.

[24] Bouchra C., Achouri M., Idrissi Hassani L.M., Hmamouchi M. (2003). Chemical composition and antifungal activity of essential oils of seven Moroccan Labiatae against *Botrytis cinerea* Pers: Fr. J. Ethnopharmacol.

[25] Tsao R., Zhou T. (2000). Antifungal activity of monoterpenoids against postharvest pathogens *Botrytis cinerea* and *Monilinia fructicola*. J. Essent. Oil Res.

[26] Mueller-Riebau F., Berger B., Yegen O. (1995). Chemical Composition and Fungitoxic Properties to Phytopathogenic Fungi of Essential Oils of Selected Aromatic Plants Growing Wild in Turkey. J. Agric. Food Chem.

[27] Martin J.A., Solla A., Witzell J., Gil L., Garcia-Vallejo M.C. (2010). Antifungal effect and reduction of *Ulmus minor* symptoms to *Ophiostoma novo-ulmi* by carvacrol and salicylic acid. Eur. J. Plant Pathol.

[28] Soković M.D., Vukojević J., Marin P.D., Brkić D.D., Vajs V., van Griensven L.J.L.D. (2009). Chemical Composition of Essential Oils of *Thymus* and *Mentha* Species and Their Antifungal Activities. Molecules.

[29] Arras G., Usai M. (2001). Fungitoxic activity of 12 essential oils against four postharvest citrus pathogens: chemical analysis of *Thymus capitatus* oil and its effect in subatmospheric pressure conditions. J. Food Protect.

[30] Caccioni D.R.L., Guizzardi M. (1994). Inhibition of germination and growth of fruit and vegetable postharvest pathogenic fungi by essential oil components. J. Essent. Oil Res.

[31] Tabanca N., Demirci B., Crockett S.L., Baser K.H.C., Wedge D.E. (2007). Chemical Composition and Antifungal Activity of *Arnica longifolia*, *Aster hesperius*, and *Chrysothamnus nauseosus* Essential Oils. J. Agric. Food Chem.

[32] Sokovic M., Griensven L.J.L.D. (2006). Antimicrobial activity of essential oils and their components against the three major pathogens of the cultivated button mushroom, *Agaricus bisporus*. Eur. J. Plant Pathol.

[33] Regnier T., Combrinck S. (2010). *In vitro* and *in vivo* screening of essential oils for the control of wet bubble disease of *Agaricus bisporus*. S. Afr. J. Bot.

[34] Alzate O., Diego A., Mier M., Gonzalo I.L., Afanador K., Durango R., Diego L., Garcia P., Carlos M. (2009). Evaluation of phytotoxicity and antifungal activity against *Colletotrichum acutatum* of essential oils of thyme (*Thymus vulgaris*), lemongrass (*Cymbopogon citratus*), and their main constituents. Vitae.

[35] Venturini M.E., Blanco D., Oria R. (2002). *In vitro* antifungal activity of several antimicrobial compounds against *Penicillium expansum*. J. Food Protect.

[36] Saddiq A.A., Khayyat S.A. (2010). Chemical and antimicrobial studies of monoterpene: Citral. Pestic. Biochem. Phys.

[37] Moleyar V., Narasimham P. (1988). Fungitoxicity of binary mixtures of citral, cinnamic aldehyde, menthol and lemon grass oil against *Aspergillus niger* and *Rhizopus stolonifer*. Lebensm. Wiss. Technol.

[38] Kishore G.K., Pande S., Harish S. (2007). Evaluation of essential oils and their components for broad-spectrum antifungal activity and control of late leaf spot and crown rot diseases in peanut. Plant Dis.

[39] Lee H.-C., Cheng S.-S., Chang S.-T. (2005). Antifungal property of the essential oils and their constituents from *Cinnamomum osmophloeum* leaf against tree pathogenic fungi. J. Sci. Food Agric.

[40] Wuryatmo E., Klieber A., Scott E.S. (2003). Inhibition of Citrus Postharvest Pathogens by Vapor of Citral and Related Compounds in Culture. J. Agric. Food Chem.

[41] Luo M., Jiang L.-K., Zou G.-L. (2002). The mechanism of loss of germination ability of *A. flavus* spore with citral. *Zhongguo Shengwu Huaxue Yu Fenzi Shengwu Xuebao* 2002, *18*, 227–233. Chem. Abs.

[42] Kurita N., Miyaji M., Kurane R., Takahara Y., Ichimura K. (1979). Antifungal activity and molecular orbital energies of aldehyde compounds from oils of higher plants. Agric. Biol. Chem.

[43] Krupa S., Nylund J.E. (1972). Ectomycorrhizae of pine. III. Growth inhibition of two root pathogenic fungi by volatile organic constituents of ectomycorrhizal root systems of *Pinus sylvestris*. Eur. J. Forest. Pathol.

